# Self-collected first-void urine cytology as a non-invasive triage tool for endometrial cancer detection

**DOI:** 10.3389/fonc.2026.1910651

**Published:** 2026-10-22

**Authors:** Jean-Ellen Johnson, Nadira Narine, Aditi R. Chandraya, Helena O’Flynn, Molly Dore, Emma J. Davidson

**Affiliations:** 1 Department of Obstetrics and Gynaecology, St Mary’s Hospital, Manchester University NHS Foundation Trust, Manchester Academic Health Science Centre, Manchester, United Kingdom; 2 Gynaecological Oncology Research Group, Division of Cancer Sciences, University of Manchester, Faculty of Biology, Medicine and Health, Manchester, United Kingdom; 3 Centre for Reproductive Health, Institute for Regeneration and Repair, University of Edinburgh, Edinburgh, United Kingdom; 4 CRUK Scotland Centre, Institute of Genetics and Cancer, University of Edinburgh, Edinburgh, United Kingdom

**Keywords:** Delphi screener, diagnostic accuracy, endometrial cancer, non-invasive, postmenopausal bleeding (PMB), triage tool, urine cytology, vaginal cytology

## Abstract

**Introduction:**

Sequential tests that measure, visualise and sample the endometrium are currently used to confirm disease status in women with suspected endometrial cancer. These tests are invasive and unpleasant, with moderate diagnostic accuracy. A non-invasive test that accurately triages women with postmenopausal bleeding (PMB) for routine diagnostics would improve patient care. The aim of this study was to measure the potential for self-collected first-void urine cytology to detect endometrial cancer in women with PMB.

**Methods:**

This was a prospective diagnostic accuracy study of women referred with suspected endometrial cancer to St Mary’s Hospital in Manchester. Self-collected first-void urine (10 mL Colli-Pee^™^ device with preservative for the first 94 participants, 20 mL with preservative thereafter) and clinician-collected vaginal samples (Delphi screener) were obtained prior to routine diagnostics. Cytology was evaluated by a cytologist blinded to participant disease status. The primary outcome was the sensitivity, specificity and accuracy of self-collected first-void urine cytology compared to vaginal cytology for endometrial cancer detection. Endometrial histopathology was the reference standard when performed; disease-negative status was defined by discharge from the cancer exclusion pathway and no cancer diagnosis at 6 months’ follow up.

**Results:**

In 200 women with PMB (102 endometrial cancer cases; 98 cancer-free controls), self-collected first-void urine cytology demonstrated a sensitivity of 73.3% (95% CI 63.5-81.6) and specificity of 90.7% (95% CI 83.1-95.7) for endometrial cancer detection, with an AUC of 0.85 (95% CI 0.80-0.89). Vaginal cytology showed higher sensitivity 92.2% (95% CI 85.1–96.6), lower specificity 84.4% (95% CI 75.5–91.0) and an AUC of 0.94 (95% CI 0.91-0.97). Agreement between urine and vaginal cytology was substantial (κ=0.70), with discordant results predominantly in low-risk tumours. Urine cytology had good sensitivity in high-risk disease and remained a strong independent predictor of endometrial cancer on multivariable analysis (adjusted OR 23.8). In an exploratory but underpowered comparison, increasing Colli-Pee^™^ device collection volume from 10 mL to 20 mL increased sensitivity (70% vs 74.6%, p=0.629).

**Discussion:**

Self-collected first-void urine cytology shows moderate sensitivity and high specificity for endometrial cancer detection, however its diagnostic accuracy is currently insufficiently high to recommend its clinical use as a ‘rule-out’ triage tool. The practical advantage of the Colli-Pee^™^ device, including its suitability for community-based self-collection, supports its potential role as a non-invasive sampling tool for women with suspected endometrial cancer, but more research is needed.

## Introduction

Endometrial cancer is the sixth most common cancer in women worldwide, with 420,368 cases and 97,723 deaths reported in 2022 (1). Incidence is rising globally, driven largely by ageing populations and obesity, with the steepest rise observed in women under 40 years, particularly in United States (2).

Abnormal vaginal bleeding is the hallmark symptom of endometrial cancer (3). Postmenopausal bleeding (PMB) occurs in over 90% of cases, although less than 5-10% have underlying endometrial cancer (4). In premenopausal symptomatic women, cancer risk is low at 0.33% (95% CI 0.23–0.48%, n = 29,059; 97 cases) (5). However, diagnostic delays are common due to attribution of vaginal bleeding to benign conditions, and under-recognition of risk factors such as obesity, Lynch syndrome and polycystic ovary syndrome by healthcare providers (6–8).

Whilst most endometrial cancer cases are diagnosed early with favourable outcomes, advanced disease carries a five-year survival of 20%, highlighting the critical importance of early detection (9).Current diagnostic pathways rely on invasive tests: transvaginal ultrasound (TVS), hysteroscopy and endometrial sampling, which are expensive, painful and poorly tolerated (10). TVS, the least invasive and most acceptable option for patients, is limited by low sensitivity in Black women (11), likely owing to high prevalence of fibroids and non-endometrioid subtypes, and low specificity in premenopausal women due to physiological variations in endometrial thickness during the menstrual cycle (12–14). Blind endometrial sampling may miss focal lesions with approximately 31% of samples inadequate for diagnostic reassurance, and a further 11% attempted procedures failing to access the uterine cavity (15–17). Outpatient hysteroscopy, indicated for endometrial irregularities, focal pathology and recurrent PMB, carries risks of bleeding, infection and uterine perforation. In addition, procedural pain and cervical stenosis often requiring repeated attempts under general anaesthesia (11, 18). These invasive diagnostics place a significant burden on patients and already overburdened healthcare systems. Given the low disease prevalence in symptomatic women, there is an urgent need for an accurate non-invasive triage tool to reassure the majority of women without sinister underlying pathology and fast track those at high risk of cancer for urgent diagnostic work up (19). Development of non-invasive cancer tests was ranked top research priority by clinicians and the general public in the James Lind Alliance priority setting partnership in 2019 (20).

Urine is an attractive biofluid for endometrial cancer detection. Studies have demonstrated that endometrial cancer biomarkers shed through the cervix into the lower genital tract, contaminating urine (21–23). A proof-of-principle study showed that combined urine and vaginal cytology in symptomatic postmenopausal women could detect endometrial cancer with a sensitivity of 91.7%, and a negative predictive value (NPV) of 91.4% (24). In a larger study of 1,836 symptomatic postmenopausal women, urine and vaginal cytology collected using standard urine collection pot showed sensitivities of 74% and 81%, and specificities of 95% and 94% for endometrial cancer detection, respectively (25). However, conventional urine specimen pots are not fit for community-based collection: they are difficult to use, collect non-standardised volumes and lack preservative, which predisposes samples to bacterial overgrowth, compromising test reliability (26, 27). To enable effective use of urine-based diagnostics, optimisation of collection methods, processing protocols and standardised testing is required.

The Colli-Pee^™^ urine collection device addresses the limitations of urine sampling by reliably collecting first void urine (FVU), which contains the highest concentration of uterine debris contaminants (28, 29), in a standardised volume and immediately mixes it with preservative to maintain sample integrity. It is user friendly, suitable for home-based collection, and demonstrates high patient acceptability and strong diagnostic performance in HPV-based cervical cancer screening (30–32). However, its potential utility for endometrial cancer detection has not been explored.

We hypothesised that FVU collect using the Colli-Pee^™^ device would optimise urine sampling for cytology-based endometrial cancer detection and achieve a higher sensitivity than is achieved with conventional urine collection in a standard specimen pot. The primary aim of this study was to measure the diagnostic accuracy of FVU urine cytology for endometrial cancer detection in symptomatic cases and controls. The secondary aim was to compare its performance with matched clinician-collected vaginal cytology to inform development of a new non-invasive triage tool for women presenting with PMB.

## Methods

### Study design and participants

This was a single-blinded prospective study conducted at St Mary’s hospital, Manchester University NHS Foundation Trust between January 2023 and July 2025. The study was approved by North West Greater Manchester Research Ethics Committee (16/NW/0660). Potentially eligible participants were identified by reviewing gynaecology cancer exclusion clinic and North West Gynaecological Oncology Multidisciplinary Team meeting lists. We included women and people with a uterus who were referred on a suspected endometrial cancer pathway with PMB who provided written informed consent to participate. We enriched for our cohort with equal numbers of known endometrial cancer cases. We excluded people who had undergone hysterectomy, previous treatment for endometrial cancer, current/previous users of the levonorgestrel intrauterine system within the past three months and those with an indwelling urinary catheter.

### Clinical data

We recorded demographic and baseline clinical data, including age, body mass index (BMI), parity, ethnicity, age at menopause, bleeding profile (duration, recurrence, volume), relevant medical history (polycystic ovary syndrome, Lynch syndrome, hypertension), current and previous use of hormonal contraception and hormone replacement therapy (HRT).

### Routine diagnostics

Women with PMB were investigated by TVS, hysteroscopy, and/or endometrial biopsy according to routine clinical guidelines. TVS was performed in all participants, with an endometrial thickness of ≥4 mm prompting outpatient endometrial sampling with a suction aspirator (pipelle). Hysteroscopy with targeted biopsy was performed when focal pathology was suspected or in cases of recurrent PMB. Endometrial biopsies and hysterectomy specimens were processed according to routine histopathology protocols and reviewed by local pathologists, with cancer cases reviewed by specialist gynaecological pathologists.

### Pathology data

Histological parameters, specifically tumour grade, depth of myometrial invasion, lymphovascular space invasion and molecular classification were retrieved from MFT pathological reports. Stage was assigned according to FIGO 2009 criteria, using hysterectomy specimens where available or cross-sectional imaging (CT or MRI) when hysterectomy had not been performed. Full molecular classification (*POLE*, MMR, p53, NSMP) was performed on all specimens from November 2024 onwards; prior to this, *POLE* testing was undertaken in line with British Association of Gynaecological Pathologists (BAGP) testing guidance (33). All testing was undertaken at MFT using standard protocols: *POLE* exonuclease domain mutations were assessed by next-generation sequencing (NGS); mismatch repair (MMR) status was determined by immunohistochemistry (IHC) for MLH1, MSH2, MSH6, and PMS2, with *MLH1* promoter methylation testing performed in cases showing MLH1 or MLH1/PMS2 loss; and p53 status was assessed by IHC, with abnormal staining patterns classified as p53-abnormal. Tumours negative for *POLE* mutation, MMR deficiency, and p53 abnormality were classified as no specific molecular profile (NSMP).

### Research sample acquisition

Research samples were collected before routine diagnostic procedures in controls. In participants with known endometrial cancer, research samples were collected ≥7 days after diagnostic procedures to minimise potential contamination by dislodged endometrial cells following routine diagnostics. Urine was self-collected first by participants at least one hour after last urination using the 10 mL or 20 mL Colli-Pee^®^ device containing urine conservation medium (Novosanis, Wijnegem, Belgium). To assess the impact of collection volume on test accuracy, a 10mL Colli-Pee^™^ device was used for the first 94 participants and a 20mL device thereafter. Vaginal samples were clinician-collected second, using the Delphi screener [Rovers Medical devices, Netherlands (34)], and immediately mixed with an equal volume of CytoRich™ Red preservative to prevent degradation and bacterial overgrowth, and preserve cellular integrity.

### Laboratory analysis

Urine and vaginal samples were transferred to the MFT Cytology Laboratory, where they were centrifuged at 3,000 RPM for 5 minutes, the supernatant decanted and the cellular pellet resuspended in 6mL CytoRich™ Red (Becton, Dickinson, USA). After 1 hour of fixation, the samples were centrifuged at 1,500 RPM for 10 minutes, the supernatant decanted and an aliquot of the pellet prepared onto a slide by centrifuging for 10 minutes at 1,500 RPM using the Hetich Rotofix 32A cytochamber centrifuge. Cytology slides were fixed in industrial methylated spirit for 10 minutes and stained with the Papanicolaou stain, then cleared in two changes of xylene and cover-slipped. All slides were screened by a Consultant Biomedical Scientist in Cytopathology using the classification system shown in **Table 1** (24).

**Table 1:** Cytology classification system.

Class	Description	Morphology	Interpretation
1	Negative	No malignant cells seen (NMCS)	Negative cytology
2	Atypical glandular cells	Glandular cells present	Positive cytology
3	Atypical cells of uncertain significance (ACUS)	Atypical cells present	Positive cytology
4	Suspicious for malignancy	Atypical glandular cells suspicious for endometrial cancer present	Positive cytology
5	Adenocarcinoma	Adenocarcinoma cells present	Positive cytology
6	Unsatisfactory sample	Sample obscured by debris, lymphocytes, bacteria or paucicellular sample	Unsatisfactory

### Reference standard

Endometrial cancer was confirmed by histological examination of endometrial biopsy samples, which served as the reference standard. For women with PMB who did not undergo endometrial biopsy, disease-negative status was defined as a normal pelvic ultrasound (endometrial thickness < 4mm) and/or normal hysteroscopy and no subsequent endometrial cancer diagnosis within six months, verified by review of clinical records.

### Sample size

A pragmatic sample of 100 treatment-naïve people with histologically confirmed endometrial cancer (cases) and 100 referred on a suspected endometrial cancer pathway with PMB (controls) were recruited. According to Bujang et al (35), 100 participants in each group is sufficient to evaluate test accuracy with acceptable precision, yielding 95% confidence intervals (CI) of +/- 10% when diagnostic performance falls within a clinically relevant range (70-90%).

### Statistical analysis

Statistical analysis was performed using SPSS version 29, IBM Corp and R version 4.5.1. Normality of continuous variables was assessed using the Shapiro–Wilk and Kolmogorov–Smirnov tests, which demonstrated non-normal distrib utions. Descriptive analyses were therefore undertaken using the Mann–Whitney U and Kruskal–Wallis tests for continuous variables, and Pearson’s chi-square or Fisher’s exact test for categorical variables, as appropriate. The primary outcome was the diagnostic accuracy of FVU urine cytology against the mixed reference standard (histology or discharge from diagnostic pathway), with sensitivity, specificity, accuracy, likelihood ratios (LR), and 95% confidence intervals (CIs) reported. To estimate predictive performance in real-world PMB populations, bayes’ theorem was used to model expected positive predictive value (PPV) and negative predictive value (NPV) at endometrial cancer prevalence of 5% and 10%, using the sensitivity (73.3%) and specificity (90.7%) observed in the study cohort. ROC curves were generated for urine and vaginal cytology, and the area under the curve (AUC) with 95% CIs estimated; AUCs were compared using DeLong’s test for paired ROC curves. Concordance and discordance between urine and vaginal cytology were examined using cross-tabulations. Agreement was quantified with Cohen’s kappa statistic, with 95% CIs and interpretation according to Landis and Koch benchmarks.

Secondary outcome measures included comparison of paired urine and vaginal cytology sensitivity and specificity, assessed using McNemar’s test, with absolute differences and 95% CIs reported. We also undertook exploratory analysis to assess the effect of urine collection volume (10 mL vs 20 mL) by stratifying accuracy estimates and comparing groups using two-proportion Z-tests. Univariate logistic regression models were used to identify candidate predictors of endometrial cancer. Variables with p<0.1, as well as those of clinical relevance, were entered into multivariable logistic regression models to evaluate whether urine and vaginal cytology provided independent discrimination after adjustment for covariates. Inadequate and or unsatisfactory cytology samples and missing data were excluded from the analysis.

## Results

### Study population

From 320 potential participants, 120 were ineligible for the following reasons: managed elsewhere (n=80), no PMB (n=11), levonorgestrel intrauterine system *in situ* (n=14), received neoadjuvant therapy (n=3), died (n=2), lacked capacity to consent (n=2), declined study participation (n=8) (**Figure 1**). In total, 200 eligible women were recruited and provided matched urine and vaginal research samples. Two control participants were subsequently diagnosed with endometrial cancer and reclassified as cases. Histology confirmed endometrial cancer in 102 cases, while 98 symptomatic controls had benign pathology, unscheduled bleeding on HRT or no cause for their bleeding was found. Among the 98 symptomatic controls, 55 (56.1%) had histologically verified benign disease and 43 (43.9%) were classified as disease negative using the non-biopsy reference standard (normal TVS and/or hysteroscopy, with no cancer at 6 month follow up). Four samples (two urine, two vaginal from different participants) were inadequate for cytological assessment due to obscuring crystal, debris or lack of viable cells and were excluded from analyses.

**Figure 1:**
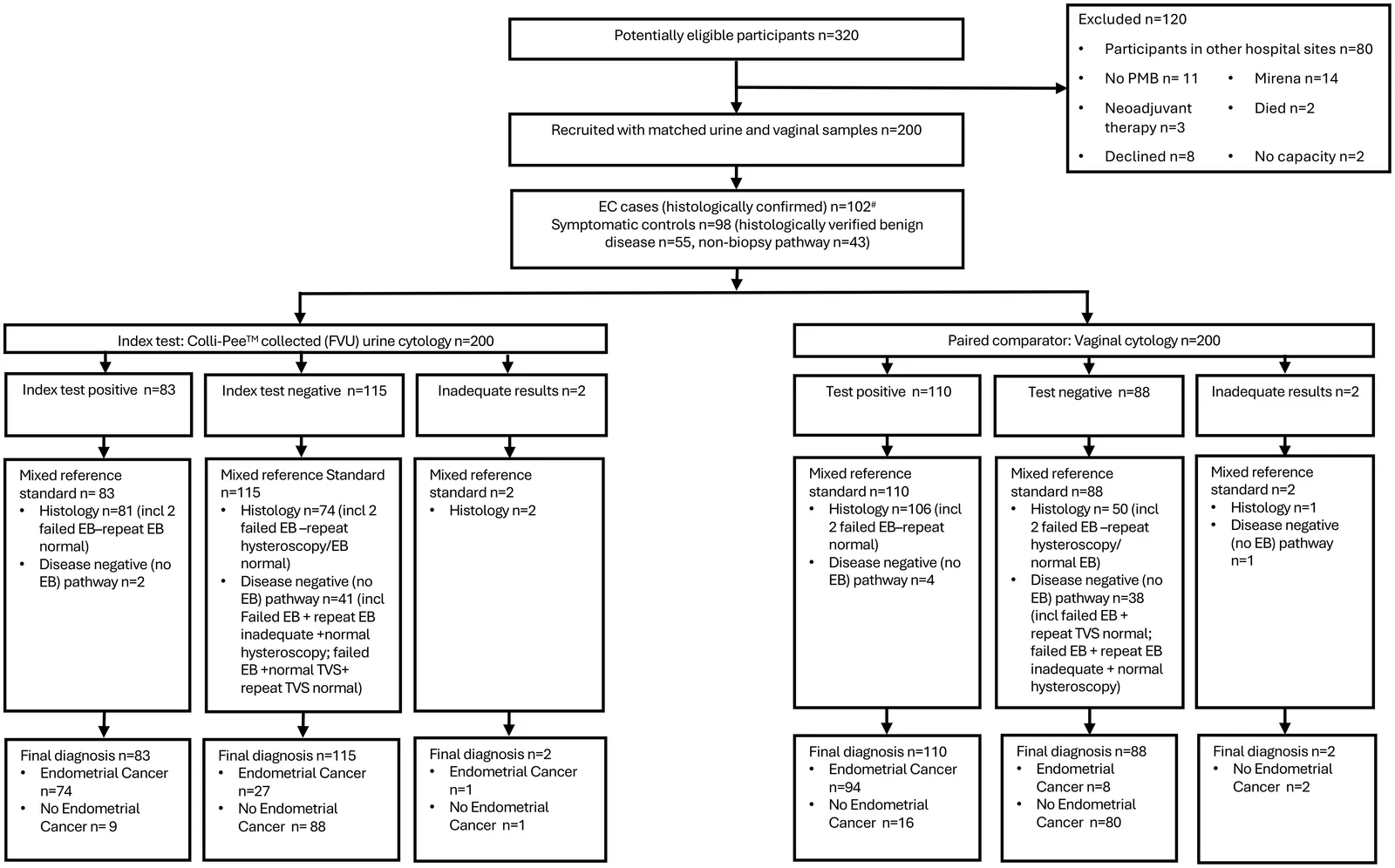
STARD study flow diagram. Index test: urine cytology (Coll-Pee); Paired comparator: vaginal cytology, Mixed reference standard: histopathology when performed; pre-specified disease negative (non-biopsy) pathway defined as (TVS 14 mm and/or normal hysteroscopy, no cancer months). Abbreviations: DC endometrial cancer; PMB postmenopausal bleeding: FVU first wold urine; RS reference standard; incl including; CD endometrial biopsy; TVS transvaginal ultrasound. Two participants who were PMB controls were subsequently diagnosed with DC and reclassified as cases.

Participants were predominantly white British (cases: 80.4% vs controls: 83.7%). Women from Asian backgrounds were more represented among endometrial cancer cases than controls (9.8% vs 2.0%, p=0.999). Women with endometrial cancer were older (median age 65 vs 59 years, p=0.001), had a higher BMI (32 vs 27 kg/m^2^, p=0.001), were more likely to be nulliparous (23.5% vs 6.1%, p=0.001), reported longer durations of bleeding (>3months 51% vs 24.5%, p<0.001) and had more recurrent bleeding (81.4% vs 62.2%, p=0.001) than benign controls (**Table 2**). Benign controls were more likely to have ever used hormonal contraception (82.7% vs 52.9%, p<0.001), HRT (39.8% vs 26.5%, p=0.039), and to be current HRT users (44.9% vs 6.9%, p<0.001).

**Table 2:** Baseline characteristics of study population.

Characteristic	Category	Total population n=200	Endometrial cancer cases*n=102	PMB controls n=98	P-value
Age (years) median (IQR)		60.5 (56.8, 68.3)	65 (57, 72)	59 (56, 62)	0.001^^^
BMI kg/m^2^ median (IQR)		29 (25.3, 34.7)	32 (27, 35)	27 (24, 32)	0.001^^^
Ethnicity, n (%)	White	164 (82)	82 (80.4)	82 (83.7)	0.099^$^
	Black	13 (6.5)	5 (4.9)	8 (8.2)	
	Asian	12 (6)	10 (9.8)	2 (2)	
	South East Asian	7 (3.5)	3 (2.9)	4 (4.1)	
	Arab	1 (0.5)	0 (0)	1 (1)	
	Mixed	1 (0.5)	1 (1)	0 (0)	
	Missing	2 (1)	1 (1)	1 (1)	
Parity, n (%)	Nulliparous	30 (15)	24 (23.5)	6 (6.1%)	0.001^$^
	Multiparous	169 (84.5)	78 (76.5)	91 (92.9)	
	Missing	1 (0.5)	0 (0)	1 (1)	
Age of menopause (years) median (IQR		51 (48-53)	50 (46-53)	51 (50-53)	0.321^^^
Ever used hormonal contraceptive, n (%)	Yes	135 (67.5)	54 (52.9)	81 (82.7)	<0.001^$^
	No	65 (32.5)	48 (47.1)	17 (17.3)	
Ever use of HRT, n (%)	Yes	66 (33)	27 (26.5)	39 (39.8)	0.039^#^
	No	133 (66.5)	74 (72.5)	59 (60.2)	
	Missing	1 (0.5)	1 (1.0)	0 (0)	
Current use of HRT, n (%)	Yes	51 (25.5)	7 (6.9)	44 (44.9)	<0.001^#^
	No	149 (74.5)	95 (93.1)	54 (55.1)	
Duration of bleeding, n (%)	7 days or less	46 (23)	14 (13.7)	32 (32.7)	<0.001^#^
	1 week to 1 month	40 (20)	10 (9.8)	30 (30.6)	
	1 to 3 months	35 (17.5)	23 (22.5)	12 (12.2)	
	More than 3 months	76 (38)	52 (51)	24 (24.5)	
	Missing	3 (1.5)	3 (2.9)	0 (0)	
Amount of Bleeding, n (%)	Spotting	52 (26)	25 (24.5)	27 (27.6)	
	Light bleed	72 (36)	35 (34.5)	37 (37.8)	0.838^#^
	Heavy bleed	73 (36.5)	39 (38.2)	34 (34.7)	
	Missing	3 (1.5)	3 (2.9)	0 (0)	
Recurrent bleeding, n (%)	Yes	144 (72)	83 (81.4)	61 (62.2)	0.001^#^
	No	51(25.5)	16 (15.7)	35 (35.7)	
	Missing	5 (2.5)	3 (2.9)	2 (2)	
Type 2 Diabetes, n (%)	Yes	32 (16)	20 (19.6)	12 (12.2)	0.179^#^
	No	167 (83.5)	81 (79.4)	86 (87.8)	
	Missing	1 (0.5)	1 (1)	0 (0)	
Hypertension, n (%)	Yes	79 (39.5)	40 (39.2)	39 (39.8)	0.933^#^
	No	121 (60.5)	62 (60.8)	59 (60.2)	
PCOS, n (%)	Yes	2 (1)	2 (2)	0 (0)	
	No	198 (99)	100 (98)	98(100)	
Lynch syndrome, n (%)	Yes	2 (1)	2 (2)	0 (0)	
	No	198 (99)	100 (98)	98 (100)	

*Two cases with PMB diagnosed with endometrial cancer.

^^^Mann-Whitney;.

^$^Fishers exact test;.

^#^Chi-squared.

PMB, postmenopausal bleeding; BMI, body mass index; HRT, Hormone replacement therapy; PCOS, polycystic ovary syndrome.

Most endometrial cancers were FIGO stage I (72/102, 70.6%) endometrioid tumours (71/102, 69.6%) (**Table 3**). In participants with complete molecular testing (n=53/102, 52%), tumours were classified as *POLE*-ultramutated (1/53, 1.9%), MMR-deficient (17/53, 32.1**%**), p53-abnormal (20/53, 37.7%) and NSMP (15/53, 28.3%). Among symptomatic controls (n=98), the most frequent diagnosis was unscheduled bleeding on HRT (27/98, 27.6%), followed by benign endometrial polyps (14/98, 14.3%). No pathological cause could be identified in 48 women (49%).

**Table 3:** Final diagnoses of cases and controls.

PMB controls		n=98 (%)
Unscheduled bleeding on HRT		27 (27.6)
Endometrial polyp		14 (14.3)
Vulvovaginal atrophy		4 (4.1)
Other benign pathology		4 (4.1)
Hyperplasia with atypia		1 (1)
No cause identified		48 (49)
Endometrial cancer cases		n=102 (%)
Histological subtype	Endometrioid	71 (69.6)
	Carcinosarcoma	5 (4.9)
	Clear cell	6 (5.9)
	Serous	14 (13.7)
	Mixed	6 (5.9)
Grade	1	39 (38.2)
	2	21 (20.6)
	3	42 (41.2)
FIGO Stage	I	72 (70.6)
	II	8 (7.8)
	III	18 (17.6)
	IV	3 (2.9)
	Missing	1 (1)
Lymphovascular space invasion	Absent	48 (47.1)
	Present	50 (49)
	Missing	4 (3.9)
Depth of myometrial invasion	<50%	55 (53.9)
	>50%	44 (43.1)
	Missing	3 (2.9)
Molecular profile of endometrial cancer cases		n=102 (%)
*POLE* (NGS)	No PV	52 (51)
	PV detected	1 (1)
	Missing	49 (48)
MMR status (IHC)	MMR proficient	70 (68.6)
	MMR deficient	30 (29.4)
	Missing	2 (2)
p53 status (IHC)	p53 abnormal	31(30.4)
	p53 wildtype	69 (67.6)
	Missing	2 (2)
Molecular classification of endometrial cancer cases (ProMisE)* n=53 (%)	*POLE* ultramutated	1 (1.9)
	MMR deficient	17 (32.1)
	p53 abnormal	20 (37.7)
	NSMP	15 (28.3)
		15 (28.3)

*Participants with full molecular testing. FIGO, International Federation of Gynaecology and Obstetrics; POLE, DNA polymerase epsilon; NGS, next generation sequencing; MMR, mismatch repair; IHC, immunohistochemistry; NSMP, no specific molecular profile; PV, pathogenic variant; ProMisE, Proactive Molecular Risk Classifier for Endometrial Cancer.

### Diagnostic accuracy of urine and vaginal cytology for endometrial cancer detection

Malignant cells were identified based on morphological features including an increased nuclear to cytoplasmic ratio, vacuolated cytoplasm, abnormal distribution of nuclear chromatin and a prominent enlarged nucleolus (**Figure 2**). Urine and vaginal cytology results were graded according to predefined criteria (**Table 4**).

**Figure 2:**
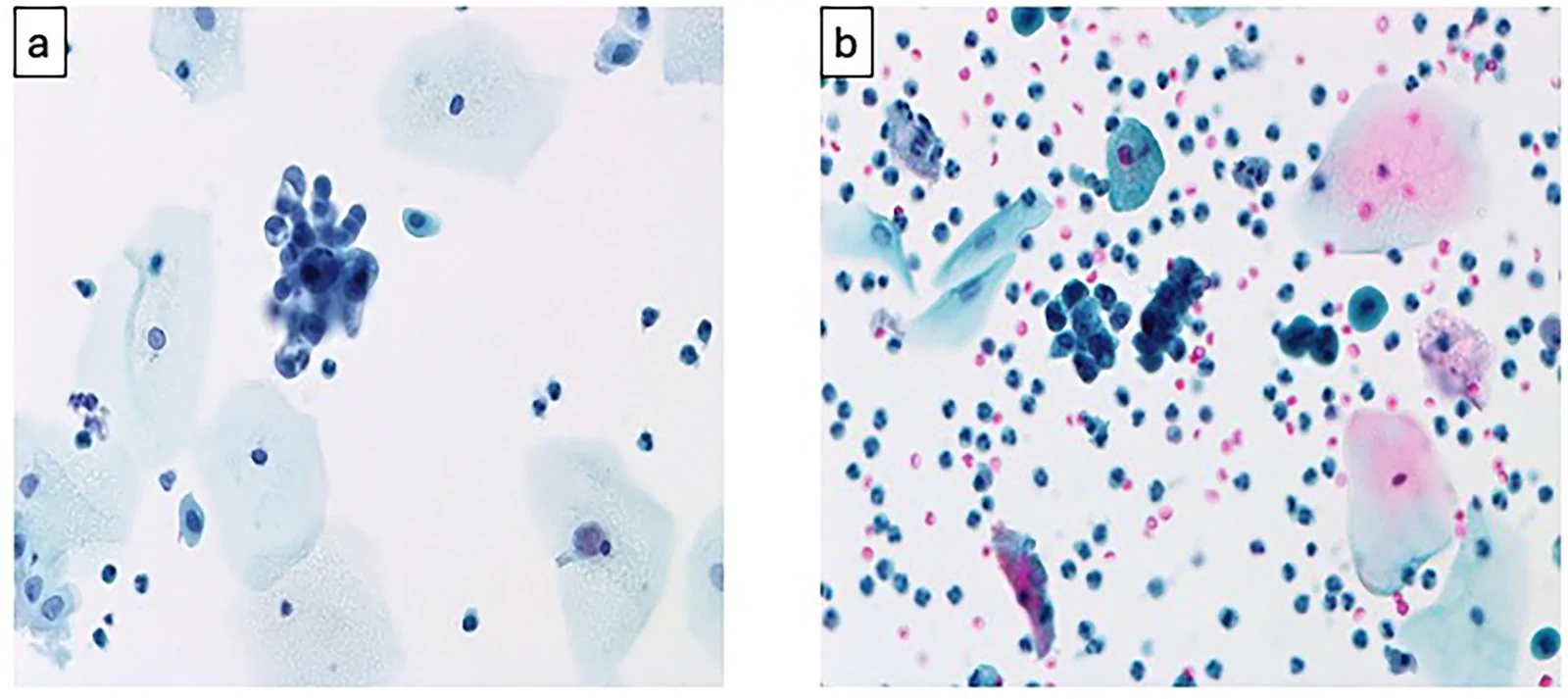
Cytology slides (Papanicolaou stain, x 400) from vaginal **(a)** and urine samples **(b)**. Cytological outcome: true positive. Clinical outcome Grade 1, Stage IB endometriold adenocarcinoma. Cytological analysis shows **(a)** a single cluster of malignant cells with a background of benign squamous cells and inflammatory cells; and **(b)** two clusters of adenocarcinoma cells on a background of benign squamous cells, neutrophils and red blood cells.

**Table 4:** Urine and vaginal cytology results.

Cytological classification	Urine cytology n= 198*	Vaginal cytology n=198*
Negative (NMCS), n (%)	115 (58.1)	89 (44.9)
Atypical glandular cells, n (%)	1 (0.5)	1 (0.5)
Atypical cells of uncertain significance (ACUS), n (%)	13 (6.6)	11 (5.6)
Suspicious for malignancy, n (%)	20 (10.1)	21 (10.6)
Adenocarcinoma, n (%)	49 (24.7)	76 (38.4)

*Inadequate results: urine sample n=2, vaginal sample n=2.

Urine cytology demonstrated a sensitivity of 73.3% (95% CI 63.5-81.6) and a specificity of 90.7% (95% CI 83.1-95.7) for endometrial cancer detection, with an overall accuracy of 81.8% (95% CI 75.7-86.9) (**Table 5**). Vaginal cytology showed significantly higher sensitivity than urine cytology at 92.2% (95% CI 85.1–96.6, McNemar’s χ^2^=17.05, p<0.001) but lower specificity at 84.4% (95% CI 75.5–91.0), however this did not reach statistical significance (McNemar’s χ^2^=3.27, p=0.070). The overall accuracy of vaginal cytology was higher than urine cytology at 88.4% (95% CI 83.1–92.5). The area under the curve (AUC) was significantly higher for vaginal than for urine cytology (0.94 (95% CI 0.91-0.97) vs 0.85 (95% CI 0.80-0.89), z=–4.57, p<0.001). The positive likelihood ratio (LR+) was higher for urine cytology than vaginal cytology 7.9 (95% CI 4.2–14.9) vs 5.47 (95% CI 3.35–8.23). Conversely, the negative likelihood ratio (LR–) was lower for vaginal cytology (0.10, 95% CI 0.06–0.21) than urine cytology (0.30, 95% CI 0.21–0.41).

**Table 5:** Diagnostic accuracy of urine and vaginal cytology for endometrial cancer detection.

Metric	Urine n=198* % (95% CI)	Vaginal fluid n=198* % (95% CI)	Test statistic	P value
Sensitivity	73.3 (63.5–81.6)	92.2 (85.1–96.6)	X^2^ = 17.05^^^	<0.001^^^
Specificity	90.7 (83.1–95.7)	84.4 (75.5–91.0)	X^2^ = 3.27^^^	0.070^^^
AUC	85 (80.0–89.4)	94 (91.1–97.4)	z=-4.64^+^	<0.001^+^
PPV	89.16 (80.4–94.9)	86.2 (78.3–92.1)	–	–
NPV	76.52(67.7–83.9)	91.0 (83.1–96.0)	–	–
Overall accuracy	81.8 (75.7–86.9)	88.4 (83.1–92.5)	–	–
Positive likelihood ratio (LR+)	7.9 (4.2–14.9)	5.47 (3.35–8.23)	–	–
Negative likelihood ratio (LR-)	0.30 (0.21–0.41)	0.10 (0.06–0.21)	–	–

Prevalence of endometrial cancer-52%.

*inadequate results: urine sample n=2, vaginal sample n=2.

^^^McNemar’s test was applied to sensitivity and specificity; + AUC comparison between urine and vaginal cytology performed using DeLong’s test. AUC, area under the curve; CI, confidence interval; PPV, positive predictive value; NPV, negative predictive value; LR+, positive likelihood ratio; LR-, negative likelihood ratio.

As the study cohort was enriched for endometrial cancer cases, predictive values were modelled at disease prevalences which are representative of routine PMB populations. At an assumed endometrial cancer prevalence of 5% and 10%, the estimated PPV was 29.3% and 46.7% and the estimated NPV was 98.5% and 96.8% respectively (**Table 6**).

**Table 6:** Modeled Predictive values of Urine cytology across real-world PMB prevalence.

Population	Prevalence (%)	Sensitivity (%)	Specificity (%)	Positive predictive value (%)	Negative predictive value (%)
Study cohort	51	73.3	90.7	89.5	76.5
Modelled PMB population	5	73.3	90.7	29.3	98.5
Modelled PMB population	10	73.3	90.7	46.7	96.8

Modelled predictive values were calculated using bayes’ theorem at assumed endometrial cancer prevalence of 5% and 10%, holding the observed cohort sensitivity 73.3% and specificity 90.7% constant. These estimates assume that diagnostic sensitivity and specificity are transferable to the real-world PMB population.

### Diagnostic accuracy of urine and vaginal cytology according to clinical and tumour characteristics

The diagnostic accuracy of urine and vaginal cytology varied with clinical and tumour characteristics. The sensitivity of urine cytology increased with age, duration of symptoms and in never versus ever users of HRT (**Table 7**). Sensitivity was highest for high grade, advanced stage tumours with poor prognostic risk factors, including p53-abnormal molecular subgroup (n=31), deep myometrial invasion (n=43) and LVSI (n=50) (**Table 8**).

**Table 7:** Diagnostic performance of urine and vaginal cytology by clinical characteristics.

Characteristic	Metric	Urine cytology % (95% CI)	Vaginal cytology % (95% CI)
Age < 60 years	Sensitivity	65.7 (47.8–80.9)	88.9 (73.9–96.9)
	Specificity	98.1 (90.1–100)	92.6 (82.1–97.9)
Age ≥ 60 years	Sensitivity	75.8 (65.6–85.5)	93.9 (85.2–98.3)
	Specificity	82.5 (67.2–92.7)	74.4 (57.9–87.0)
BMI (<30kg/m^2^)	Sensitivity	71.1 (54.1–84.6)	89.7 (75.8–97.1)
	Specificity	93.0 (83.0–98.1)	87.5 (75.9–94.8)
BMI (≥30kg/m^2^)	Sensitivity	72.9 (59.7–83.6)	93.2 (83.5–98.1)
	Specificity	83.7 (69.3–96.2)	80.0 (61.4–92.3)
Nulliparous	Sensitivity	76.0 (54.9–90.6)	92.0 (74.0–99)
	Specificity	100.0 (47.8–100)	80.0 (29.9–98.9)
Multiparous	Sensitivity	71.4 (59.8–80.9)	92.3 (84.0–97.1)
	Specificity	89.8 (81.0–94.9)	85.1(75.8–91.8)
No current HRT use	Sensitivity	73.4 (63.3–82)	92.6 (85.4–97)
	Specificity	86.5 (74.2–94.4)	73.6 (59.7–84.7)
Current HRT use	Sensitivity	62.5 (25.9–89.8)	87.5 (46.7–99.3)
	Specificity	97.6 (87.4.–99.9)	100.0 (91.2–100)
<3 months bleeding	Sensitivity	64.6 (49.5–77.8)	93.9 (83.1–98.7)
	Specificity	92.9 (84.1–97.6)	85.7 (75.3–92.9)
≥3 months bleeding	Sensitivity	80.4 (66.9–90.2)	90.2 (78.6–96.7)
	Specificity	87.5 (67.6–97.3)	82.6 (61.2–95.0)

BMI, body mass index; HRT, hormone replacement therapy; CI, confidence interval.

**Table 8:** Diagnostic performance of urine and vaginal cytology by tumour characteristics.

Characteristic	Metric	Urine cytology % (95% CI)	Vaginal cytology % (95% CI)
Low grade, n= 60	Sensitivity	67.8 (54.4–79.4)	91.7 (81.6–97.2)
	Specificity	91.5 (83.9–96.3)	84.9 (76.0–91.5)
High grade, n=42	Sensitivity	80.5 (65.1–91.2)	92.7 (80.1–98.5)
	Specificity	91.5 (83.9–96.3)	84.9 (76.0–91.5)
Early stage, n=79	Sensitivity	68.8 (57.3–78.9)	92.3 (84.0–97.1)
	Specificity	91.5 (83.9–96.3)	84.9 (76.0–91.5)
Late stage, n=21	Sensitivity	90.5 (69.6–98.8)	95.2 (76.2–99.9)
	Specificity	91.5 (83.9–96.3)	84.9 (76.0–91.5)
<50% MI, n=54	Sensitivity	63.0 (48.7–75.7)	90.9 (80.0–97.0)
	Specificity	91.5 (83.9–96.3)	84.9 (76.0–91.5)
≥50% MI, n=43	Sensitivity	88.4 (74.9–96.1)	97.7 (87.7–99.9)
	Specificity	91.5 (83.9–96.3)	84.9 (76.0–91.5)
LVSI absent, n=46	Sensitivity	58.7 (43.2–73.0)	89.4 (76.9–96.5)
	Specificity	91.5 (83.9–96.3)	84.9 (76.0–91.5)
LVSI present, n=50	Sensitivity	88.0 (75.7–95.5)	98.0 (89.4–99.9)
	Specificity	91.5 (83.9–96.3)	84.9 (76.0–91.5)
Endometrioid, n=72	Sensitivity	68.6 (56.4–79.1)	91.5 (82.5–96.8)
	Specificity	91.5 (83.9–96.3)	84.9 (76.0–91.5)
Non-endometrioid, n=30	Sensitivity	83.3 (65.3–94.4)	93.3 (77.9–99.2)
	Specificity	91.5 (83.9–96.3)	84.9 (76.0–91.5)
*POLE* mutant*, n=1	Sensitivity	100 (2.5–100)	100 (2.5–100)
	Specificity	91.5 (83.9–96.3)	84.9 (76.0–91.5)
dMMR, n=30	Sensitivity	73.3 (54.1–87.7)	93.3 (77.9–99.2)
	Specificity	91.5 (83.9–96.3)	84.9 (76.0–91.5)
P53abn, n=31	Sensitivity	80.6 (62.5–92.5)	93.5 (78.6–99.2)
	Specificity	91.5 (83.9–96.3)	84.9 (76.0–91.5)
NSMP^#^, n=14	Sensitivity	64.3 (38.8–83.7)	85.7 (57.2–98.2)
	Specificity	91.5 (84.1–95.6)	84.9 (76.3–90.8)

BMI, body mass index; HRT, hormone replacement therapy; MI, myometrial invasion; CI, confidence interval; p53abn, p53–abnormal; *POLE*, DNA polymerase epsilon; dMMR, mismatch repair deficient. *only one participant was *POLE* mutant; NSMP, no specific molecular profile, #participants with a complete molecular profile.

### Predictors of endometrial cancer

In univariable analyses, urine and vaginal cytology were strongly associated with endometrial cancer (urine cytology OR 30.38, 95% CI 12.77–72.27, p <0.001; vaginal cytology OR 57.13, 95% CI 22.72–144.70, p <0.001). Every year of age increased the odds of endometrial cancer by approximately 5% (OR 1.05, 95% CI 1.02–1.09, p=0.005) and every 1 kg/m^2^ increase in BMI increased the odds of endometrial cancer by a similar margin (OR 1.05, 95% CI 1.01–1.10, p=0.018). Prolonged bleeding (≥3 months; OR 3.35, 95% CI 1.82–6.14, p <0.001) and recurrent bleeding (OR 2.94, 95% CI 1.49–5.79, p= 0.002) were associated with higher odds of endometrial cancer whilst multiparity (OR 0.21, 95% CI 0.08–0.54, p=0.001), ever use of hormonal contraception (OR 0.23, 95% CI 0.12–0.45, p <0.001) and ever use of HRT (OR 0.23, 95% CI 0.13–0.42, p <0.001) were associated with lower odds.

In the multivariable model, after adjusting for age, BMI, parity, ever use of hormonal contraception, HRT use, duration of bleeding and recurrent bleeding, positive cytology remained an independent predictor of endometrial cancer (urine adjusted OR 23.78, 95% CI 8.29–68.15, p<0.001; vaginal aOR 38.9, 95% CI 12.57–120.68, p<0.001). Among the independent clinical variables in the model, multiparity (aOR 0.17, 95% CI 0.04-0.82, p=0.028), ever use of hormonal contraception (aOR 0.35, 95% CI 0.12–0.95; p=0.040) and current HRT use (aOR 0.20, 95% CI 0.06-0.72; p=0.013) were associated with reduced odds, while recurrent bleeding (aOR 3.58, 95% CI 1.09-11.78; p=0.036) was associated with increased odds of endometrial cancer.

### Concordance of urine and vaginal cytology

Among 102 endometrial cancer cases, urine and vaginal cytology were concordant (both positive) in 74/102 cases (73.3%), while vaginal cytology detected 19/102 (18.8%) cases missed by urine cytology. No cases were detected by urine cytology alone. Of the two women from the PMB cohort who were subsequently diagnosed with endometrial cancer, one case was positive on both urine and vaginal cytology, while the other case was negative on both tests. Overall agreement between urine and vaginal cytology was substantial (κ=0.70, 95% CI 0.60–0.79).

### Urine false negative cytology results

Across 102 endometrial cancer cases with paired sampling, urine cytology yielded 27/102 (28.4%) false negatives: 19 were missed by urine only and 8 were missed by both urine and vaginal cytology. Vaginal cytology had 8/102 (7.8%) false negatives, all of which were shared misses.

Urine false negatives were more prevalent in lower risk disease: early stage (24/27, 88.9%), low grade (19/27, 70.4%), endometrioid histology (22/27, 81.5%), <50% myometrial invasion (20/27, 74.1%), absent LVSI (19/27, 70.4%). The tumour was confined to an endometrial polyp in 11/27 (40.7%) missed cases. Vaginal cytology demonstrated the same pattern by stage, invasion, LVSI and histology.

### Exploratory comparison of diagnostic performance of 10 mL versus 20 mL Colli-Pee^™^ devices for endometrial cancer detection

The Colli-Pee^™^ device was changed from 10 mL to 20 mL mid-study, to evaluate diagnostic performance by collection volume. There were no significant differences in age, ethnicity, parity, bleeding profile (duration, amount, recurrence) and comorbidities such as diabetes and hypertension between the 10mL (n=94) and 20mL cohort (n=106). Tumour characteristics including grade, stage, myometrial invasion and LVSI did not differ between groups. However, the 20 mL cohort had a significantly higher median BMI (31 vs 28 kg/m^2^, p=0.027), lower prevalence of ever use of HRT (31.1% vs 56.4%, p=0.0003), current HRT use (16.0% vs 36.2%, p=0.001) and ever use of hormonal contraception (61.3% vs 74.5%, p=0.042) and a higher proportion of endometrial cancer cases (10 mL n=30; 20 mL n=72).

Diagnostic accuracy was similar between the two collection devices. Sensitivity was higher for the 20 mL device (74.6%, 95% CI 63.0–83.0) than the 10 mL device (70%, 95% CI 52.0–83.0), although this did not reach statistical significance, likely due to small numbers (Z=-0.482, p=0.629). Specificity was higher with the 10 mL device (93.8% (95% CI 84.8–98.3) vs 84.8% (70.0–94.9; Z = 1.431, p=0.152)) but this did not reach statistical significance. The 10 mL device had a higher positive likelihood ratio (11.2, 95% CI 4.21–29.76) vs (4.75, 95% CI 2.10–10.76; z=1.32, p=0.187) whereas the negative likelihood ratios values were similar for the two devices (0.32, 95% CI 0.19–0.56 vs 0.31, 0.20–0.47; z=0.14, p=0.891).

## Discussion

In this diagnostic accuracy study, self-collected first void urine cytology showed moderate sensitivity (73.3%) and high specificity (90.7%) for endometrial cancer detection. Vaginal cytology demonstrated significantly higher sensitivity (92.2%), with slightly lower specificity (84.4%), although this did not reach statistical significance. Overall discrimination favoured vaginal cytology (AUC 0.94 vs 0.85, respectively). Agreement between urine and vaginal cytology was substantial (κ=0.70), with discordance concentrated in lower-risk tumours (early stage, low grade, <50% myometrial invasion, LVSI-negative). Urine cytology showed good sensitivity in high-risk subgroups (high grade, advanced stage, ≥50% myometrial invasion, LVSI-positive, non-endometrioid and p53-abnormal tumours), although underpowered for such comparisons. Because it missed very few cancer cases, vaginal cytology maintained high sensitivity even in low-risk disease. Urine and vaginal cytology were strong, independent predictors of endometrial cancer after adjusting for age, BMI, parity, HRT and bleeding profile (adjusted OR 23.78 and 38.9, respectively), providing useful discrimination beyond routine clinical variables. The sensitivity of self-collected first void urine cytology was higher in women with prolonged bleeding, recurrent bleeding, older age and higher BMI, and lower in current HRT users. The 20 mL Colli-Pee^™^ device showed higher sensitivity than the 10 mL device for endometrial cancer detection, but this exploratory analysis was underpowered and did not reach statistical significance.

### Interpretation in the context of existing literature

Our findings confirm and extend previous work showing that shed malignant endometrial cells can be detected in urine and vaginal samples (22, 24, 25). Self-collected first void urine cytology achieved similar diagnostic accuracy to a standard, preservative-free urine collection pot as used in the DETECT study (sensitivity 73.3% vs 74%; specificity 90.7% vs 95%), suggesting that the addition of preservative and standardised first-void collection did not translate into a measurable diagnostic gain; thus our hypothesis was not supported. Indirect comparisons should be interpreted cautiously given differences in study populations, pre-analytics and laboratory processing. Whilst the Colli-Pee^™^ device confers no diagnostic accuracy advantage over a standard urine pot for this indication, its practical benefits are meaningful: it standardises first-void collection volume, incorporates an integrated preservative to prevent bacterial overgrowth and supports home-based self-collection with postal return features that are not achievable with a standard urine specimen, enabling community-based deployment of urine cytology outside of clinical settings.

Whilst its performance for endometrial cancer detection was insufficient to recommend urine cytology as a definitive ‘rule-out’ test, this study generated some interesting hypotheses worthy of further exploration. We saw differential performance of urine cytology across high- and low-risk tumours, suggesting that tumour shed may be proportionate to tumour volume (22, 36), with smaller tumours confined to polyps less likely to have positive urine cytology. Vaginal sampling benefits from anatomical proximity to the uterine cavity and this may be why it detected lower burden disease more reliably (37). Prolonged, heavy or recurrent PMB was associated with true positive urine cytology, whilst current HRT use was associated with false negative urine cytology. A biological explanation for this could relate to the known mechanisms of action of progestin, which stabilises the endometrium, reduces endometrial shedding and causes endometrial atrophy (38–40). The apparent superior performance of urine cytology for the detection of high risk endometrial cancer may support its use as a tool to reduce health inequities experienced by women from Black ethnic backgrounds, whose predisposition towards p53 abnormal endometrial tumours poses both diagnostic challenges (13, 14, 41) and poor outcomes (3).

### Strength and limitations

Strengths of this study include the prospective design and systematic evaluation of the Colli-Pee^™^ device in a clinically relevant PMB cohort, and the use of paired vaginal cytology as a positive control, which allowed us to attribute discordance to urine-specific factors. Cytology was reported by a single experienced consultant biomedical scientist in cytology, reducing inter-observer variability. We explored clinicopathological correlates, molecular subgroups and pre-analytic factors such as collection volume, providing a detailed characterisation of where urine cytology performs well and where further optimisation is required.

This study has limitations typical of diagnostic test accuracy research. First, this study was enriched for known cancer cases and had a relatively small prospective cohort of participants with an unknown cancer status at the time of sampling. A mixed reference standard was used: histology where performed, and disease-negative status defined as discharge from the standard diagnostic pathway. This may introduce verification bias because not all participants classified as disease-negative underwent confirmatory endometrial biopsy. In the non-biopsied group, the six month follow up may have been insufficient to identify indolent or initially occult malignancy which may affect specificity. Longer follow up periods would strengthen future prospective validation. In addition, the study design resulted in an artificially high cancer prevalence (51%), so predictive values observed were not generalisable to routine PMB populations. We therefore modelled predictive numbers at 5% and 10% prevalence, although these estimates require prospective validation. Second, inadequate cytology samples were excluded from analysis; however, these accounted for only 2/200 (1%) urine and 2/200 (1%) vaginal samples and therefore are unlikely to affect overall estimates. Third, the single centre nature of our study and its limited diversity in terms of ethnicity, means that we cannot extrapolate its findings to other centres, healthcare settings or populations. This is important because current diagnostics perform differently in women from different backgrounds (41). Fourth, the comparison between 10mL and 20mL Colli-Pee^™^ devices was underpowered, and significant differences between the cohorts in BMI, HRT and hormonal contraception use, and the number of cancer cases represent potential confounding factors. Therefore, any observed differences in diagnostic accuracy cannot be attributed to collection volume alone. Fifth, participants recruited after a known endometrial cancer diagnosis, research sampling occurred following diagnostic procedures, although a minimum interval of 7 days was used to minimise contamination from procedure-related cellular shedding. Nevertheless, an effect of prior endometrial biopsy or hysteroscopy on subsequent cytological yield cannot be excluded and represents a potential source of bias. Finally, we tested urine cytology as a triage tool in symptomatic women and thus cannot attest to its clinical utility as a screening tool in asymptomatic women, such as those with Lynch syndrome.

### Clinical relevance

Since endometrial cancer risk in women with PMB is just 5-10% (4, 42), and even lower in current HRT users (43), there is an urgent need for accurate, non-invasive strategies to reduce the burden of unnecessary invasive diagnostics on women and healthcare services (44). The clinical challenge in PMB is balancing sensitivity (minimising missed cancers) against specificity (reducing unnecessary invasive diagnostics). As our cohort was enriched for endometrial cancer cases, we modelled expected predictive values at assumed endometrial cancer prevalences of 5% and 10% which are more representative of routine PMB populations. This increased the estimated NPV to 98.5% and 96.8% respectively, although these estimates require prospective validation in an unselected clinical cohort. Despite the high modelled NPV, the moderate sensitivity of urine cytology, particularly for low-risk tumours, means that it cannot be recommended as a standalone rule-out test. Positive urine cytology would require fast-track referral for confirmatory endometrial biopsy, diagnostic work-up and treatment. Histopathological examination of adequately sampled endometrial tissue remains the diagnostic gold standard. The potential clinical role of urine cytology may therefore be as a non-invasive, community-based preliminary triage tool, particularly where vaginal sampling is unacceptable or not feasible. Its self-collection capability may facilitate testing before hospital attendance and could support prioritisation within PMB pathways; however negative results would require appropriate safety-netting and further investigation of symptomatic women according to established diagnostic pathways.

By contrast, vaginal cytology shows excellent sensitivity for the detection of both high-risk and low-risk tumours, and as such could offer a viable, minimally-invasive triage tool for use in the community or in secondary care, to triage women with PMB for routine invasive diagnostics (25). As urine cytology did not identify any cancers missed by vaginal cytology, our findings do not support combining the two sampling approaches to improve cancer detection. Cytology is a subjective test that requires training and may not be scalable without further work on inter-observer performance and potential adjuncts such as digital image analysis or molecular assays.

For high-risk women such as those with Lynch syndrome who are subjected to repeated annual invasive tests, non-invasive urine sampling using a Colli-Pee^™^ device that enables home-based collection with postal return to the lab is a particularly attractive potential strategy. Although the observed sensitivity at the prespecified threshold is insufficient for urine cytology to serve as a standalone surveillance test, its potential performance in detecting high-risk disease suggests a role in risk-stratified pathways, where the aim is to prioritise those with the most clinically significant pathology for expedited assessment.

### Future directions

Our findings highlight urine and vaginal fluid as a versatile biofluid for the non-invasive detection of endometrial cancer. Emerging data show that genomic endometrial cancer biomarkers can be detected in urine and vaginal fluid (45–47), supporting their use for downstream molecular profiling to strengthen risk stratification (48). In parallel, there is a clear opportunity to improve scalability and reproducibility of urine and vaginal cytology through digitisation, automation and machine learning algorithms. Artificial intelligence could support automated pre-screening, slide prioritisation and improve consistency of reporting in this context (49). These developments could extend urine and vaginal fluid from a cytology-based triage tool to an integrated platform for molecular characterisation and surveillance in high risk groups, including women with Lynch syndrome.

Future work should include contemporaneous, within-participant comparisons of the Colli-Pee^™^ device and standard urine specimen pots, formal inter-observer assessments to refine cytology thresholds and narrow the performance gap with vaginal cytology. Evaluation of urine and vaginal cytology embedded within defined clinical pathways (PMB triage, Lynch syndrome gynaecological surveillance) and accompanying health-economic analyses will be essential to determine its optimal positioning. Development of molecular biomarkers in urine and vaginal fluid may ultimately offer a combined cytological-molecular platform that maximises sensitivity while preserving the practical advantages of non-invasive diagnostic strategies.

## Conclusion

Clinician-collected vaginal cytology delivers consistently high sensitivity across clinical and tumour strata and excellent overall discrimination, whereas self-collected first void urine cytology offers moderate sensitivity and high specificity for endometrial cancer detection. Urine cytology is an independent predictor of endometrial cancer and has several practical strengths: it is simple, non-invasive, acceptable, and amenable to community collection and postal return, including scenarios where vaginal sampling is not feasible (e.g., frailty, intolerance of pelvic examination). However, it cannot provide a definitive diagnosis, and its moderate sensitivity precludes its use as a standalone rule-out test. Further research is needed before urine cytology can be recommended for routine clinical care.

## Data Availability

The raw data supporting the conclusions of this article will be made available by the authors, without undue reservation.
